#  Genomes, Populations and Diseases: Ethnic Genomics and Personalized Medicine 

**Published:** 2010

**Authors:** V.A. Stepanov

**Affiliations:** Research Institute for Medical Genetics, Siberian Branch, Russian Academy of Medical Sciences

## Abstract

This review discusses the progress of ethnic genetics, the genetics of common diseases, and the concepts of personalized medicine. We show the relationship between the structure of genetic diversity in human populations and the varying frequencies of Mendelian and multifactor diseases. We also examine the population basis of pharmacogenetics and evaluate the effectiveness of pharmacotherapy, along with a review of new achievements and prospects in personalized genomics.

##  Personalized medicine 

The concept of personalized medicine, which puts the individual patient, with all of his specific peculiarities, into the center of attention, is not new. The 19th-century Russian physicians M.Y. Mudrov and N.I. Pirogov were well aware of this principle. “A doctor treats the patient, not the disease… Each patient needs special treatment depending on his physical constitution, even if the disease is the same,” wrote Mudrov. The great medical practitioners of the past also acknowledged the prophylactic value of personalized medicine – “Healthy people should be kept in hand… they should be advised on keeping to a healthy lifestyle” [1] and “a disease is easier to prevent than to treat” [2]. A new appreciation and the real potential for personalized medicine have re-appeared in the age of molecular genetics. By the end of the 1990s, a new concept of genomic medicine had started to take form [3, 4], and this concept involved “the routine use of genotyping methods, usually in the form of DNA-testing, for improving the quality of healthcare” [3]. The modern understanding of personalized medicine is based on the principles of preventive medicine, which were postulated by Nobel-prize laureate Jean Dausset [5]. The outlines of this concept are eloquently described by the 4P medicine principle or system medicine, which was suggested by Leroy Hood [6–8]. This principle states that “reactive” medicine (which reacts to a disease and fights its symptoms) must turn into predictive, preventative, personalized, and participatory medicine; namely, medicine that will be aimed at predicting the disease before it manifests itself through symptoms, take into account any individual (mainly genetic) traits of the patient, as well as involve the active help of the patient in identifying his or her genetic traits and in determining preventive measures.

In Russia, ideas related to personalized medicine based on the advances in molecular genetics are being actively pursued at several genetic schools. One school, headed by RAMS full member V.P. Puzyrev [9-11], is developing the concept of genomic medicine. Another, headed by RAMS corresponding member V.S. Baranov [12-14], is developing the concept of genetic passports.

##  Ethnic genetics 

 The geographical region, ethnic group, and population largely determine the genetic traits of an individual. Setting aside the issues of the substance, the terminological and meaningful differences in the geographic, ethnic and population levels of gene-pool organization, we shall review these terms in the context of personalized medicine in which they are essentially synonymous – they reflect the individual traits of a human that are dependent on his genetic origins. 

It is agreed that a detailed understanding of genetic diversity in human populations is crucial for determining the genetic basis of most common diseases [15]. Approaches aimed at identifying genetic similarities between various ethnic groups and populations, and which involve the study of polymorphic genetic markers, have been used in evolutionary and population genetics since the middle of the 1950s. Initially, protein polymorphisms played the role of genetic markers [16–18]. However, as molecular genetic techniques improved, population studies were reoriented toward various classes of DNA markers, with non-recombinant lineages of mtDNA and Y-chromosomes being the most widely used [19–21, etc.]. These studies allowed researchers to form an understanding of the main stages of population spread and of the ethnic divergence of modern humans. This also resulted in the appearance of a new scientific field: ethnogenetics. According to Balanovsky and Rychkov [22], ethnogenetics is a branch of population genetics which “pays special attention to the ethnic structure of populations and attempts to identify the genetic results of the ethno-historic and ecological development of human populations.”

 Currently, the most useful tools for describing genetic variability in various ethnic groups and populations are genome-wide sets of single nucleotide polymorphisms (SNPs), which are sometimes complemented by copy number variability (CNV) data [23–26, etc.]. A promising method that is sure to be used in years to come is the re-sequencing of complete genomes in representative cohorts from different populations. 

Studies in ethnogenetics are among the most productive areas of genetic science in Russia, and such studies are being actively pursued in a number of research facilities [27–31].

 The importance of population genetics for personalized medicine also derives from the fact that knowledge of the role of genetic variability in the pathogenesis of common diseases can only be obtained by a detailed analysis of the associations between genetic markers and diseases on large cohorts of patients and healthy people from various populations. Specifically, one of the most productive approaches for association analysis, the so-called Genome-wide Association Study (GWAS), requires the testing of hundreds, if not thousands, of individuals and replication of the discovered associations in other populations. 

 The cooperative development of ethnic genetics, the genetics of common diseases, and the concept of personalized medicine spawns a number of key questions. Answers to these questions will determine to what use and how quickly genetics will be adopted in predictive medicine: 

 - How marked are interethnic differences in disease incidence and disease susceptibility gene frequencies? 

 - What are the evolutional mechanisms behind the differences in disease gene frequencies? 

 - Do racial, ethnic or geographical origins influence the impact of distinct genetic variants on the course of a disease? 

 - To what degree does genetic diversity account for the differences in the spread and outcome of diseases between racial and ethnic groups? 

 - Is there any need for information on the racial/ethnic origin of patients for medical research? 

 Drawing up a picture of the current understanding of the answers to these questions is the main aim of this article. 

##  Structure of genetic variability in human populations 

How different are human populations genetically? Human population genetics give a precise answer to this question – interpopulation differences in a global sense (comparing the populations of different continents) are responsible for 10–15% of the genetic variability in humans (Table 1). In other words, the Wright Fixation Index ( *F*_st_ ) is 0.10-0.15 when estimating the global level of genetic differentiation in human populations. This interval includes values obtained for most systems of genetic markers in classical and molecular population genetics of humans -- blood type, protein polymorphism, RFLP, Alu-repeats, hypervariable segments of mtDNA [28, 32]. Exceptions are the highly mutable microsatellites (STR), whose level of genetic differentiation is much lower (4–5%), and the  *Y* -chromosome, whose variants differ (20-30%) between populations much more than other marker systems. These two types of markers are so distinctly different because of specific evolutional, population, and social mechanisms we will not discuss in this work (see [28]).

**Table 1:** Genetic Differentiation of Human Populations

Marker type	Populations	F_ST_	Reference
Classical markers			
Blood types	World	0.16	[40]
Protein polymorphism	World	0.11	[40]
DNA-markers			
RFLP	-“-	0.11	[21]
dinucleotide	-“-	0.11	[41]
trinucleotide	-“-	0.04	[42]
tetranucleotide	-“-	0.04	[21, 43]
Microsattelites and RFLP	-“-	0.15	[44]
Alu-repeats	-“-	0.12	[45]
Alu-repeats	-“-	0.10	[46]
mtDNA HVSI	-“-	0.14	[47]
Y-chromosome, haplogroups	North Eurasia	0.19	[28]
Y-chromosome STR	North Eurasia	0.19	[28]
Genome-wide marker sets			
600К SNP	YRI, CEU, JPT, CHB	0.12	[48]
1 million SNP	YRI, CEU, JPT, CHB	0.10	[48]
1 million	YRI, CEU, JPT, CHB	0.13	[49]
440K SNP	World	0.05	[26]
50K SNP	Asia	0.06	[25]
2.8 million	YRI, CEU, JPT, CHB	0.11	[50]
244K SNP	World	0.12	[51]
200K SNP	World	0.13	[33]
67 CNV	World	0.11	[52]

**Fig. 1:**
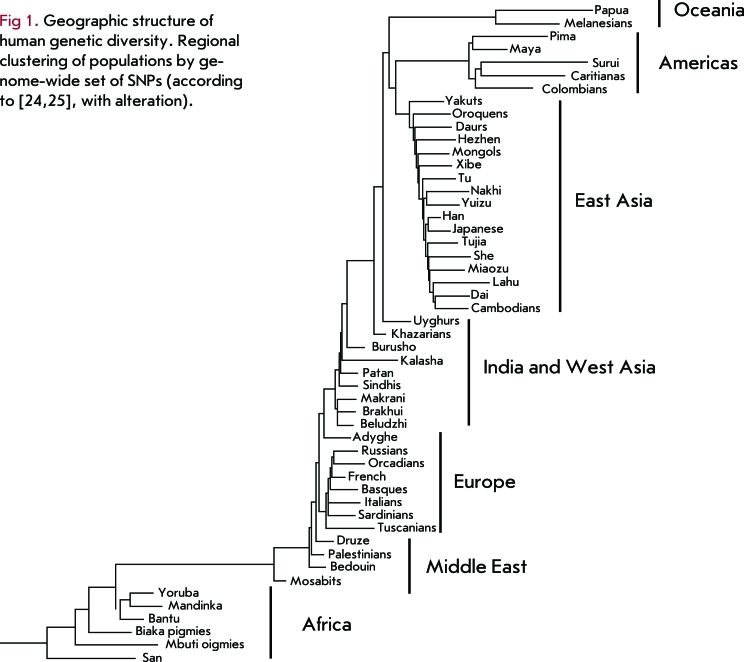
Fig 1. Geographic structure of human genetic diversity. Regional clustering of populations by genome-wide set of SNPs (according to [24, 25], with alterations).

A relatively low level of genetic subdivision in human populations can be observed in the most representative and complete sets of markers – on large and random datasets of autosomal polymorphisms, including genome-wide sets of hundreds of thousands of SNPs. Li *et al* . [24] analyzed data for 650,000 SNPs in 51 populations obtained from the Human Genome Diversity Project (HGDP) and found that interpopulation differences accounted for 11% of overall genetic diversity. Recent work by us yielded another estimate of the genetic differentiation in 36 populations (32 Eurasian populations and 4 HapMap populations) for 200,000 SNPs, the result being 13.4% [33]. Somewhat smaller genetic differences were observed during the analysis of a lower number of continental groups. The level of genetic differentiation between populations in Asia is 5.9% according to data from the Panasian SNP Consortium [25], while the population differentiation in East Asia, South Asia, Europe, and Mexico is 5.2% [26].

The low level of genetic differentiation in human populations as compared to related species (chimpanzees ( *F*_ST_ = 0.32) [34] and gorillas ( *F*_ST_ = 0.38) [35]), despite the much larger population area, indicates that the human population originated relatively recently from a small number of ancestors.

The most general distribution pattern of human population diversity is its strict geographical structure, namely the clustering of geographically adjacent populations. On a worldwide scale, populations can be grouped into racial-continental groups for any set of markers. These groups are African Negroids, Caucasoids (which are divided into the Middle Eastern, European, and Indian sub-clusters), Asian Mongoloids, Austronesians, and American Indians [24, 25, 36] (Fig. 1). This pattern can also be observed on a smaller scale - for continental and subcontinental groups of populations [23, 37]. The projection of genetic differences between representative population datasets onto a space of major components or factors always yields a geographical map at first approximation. The cause of such a distribution is the evolutionary history of genetic diversity, which resulted mainly from migration and genetic drift during the spread of modern humans.

The population of Russia is not exempt from this pattern. Russian populations cluster into several large ethnogeographical groups: Slavs, Northern Caucasus populations, Finno-Ugric peoples of the North European and Volga-Ural regions, the populations of South Siberia and Central Asia, and the populations of Eastern Siberia and North Asia [28, 33]. The geographical structure of the Russian gene pool can be observed for all of the genetic markers – lineages of mtDNA, *Y* -chromosomes, *X* -chromosomes, and autosomal markers, including complete genomic sets of SNPs.

**Fig. 2:**
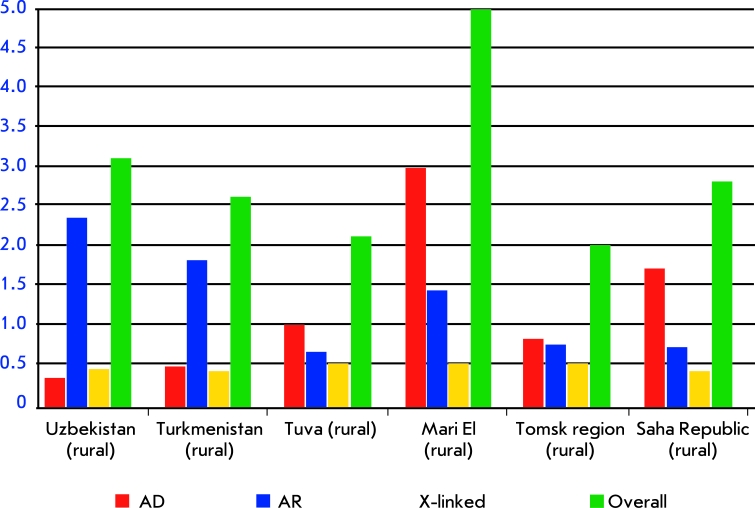
Cumulative frequency of monogenic diseases in several populations in Russia and neighboring countries.

It is probable that the only major exception to the “geographical pattern” is the Indian subcontinent, in which the genetic diversity is better correlated to the language group, rather than the geographical origin. This is due to the complex ethnic and social structure of the population, caste hierarchy of large ethnic groups, and the presence of numerous small clan/tribe groups [38, 39].

**How different are human populations in terms of disease incidence and disease gene frequency?**

 If we assume that the question of general interpopulation genetic diversity in humans has been answered, then the following questions arise: To what degree is genetic variation correlated with phenotypic variation, especially for clinical phenotypes (diseases)? To what degree are the differences in disease gene frequencies responsible for the interethnic and interpopulation differences in disease incidence? The first question can be answered using data on genetic epidemiology and medical statistics, while the second question needs special approaches. 

**Ethnic component of monogenic diseases**

Genetic epidemiology has collected a large set of data on the frequency of Mendelian (monogenic) diseases in various populations. The overall load of hereditary diseases (HD) (the summed frequencies of autosomal dominant, autosomal recessive and X-linked diseases) in stable populations is relatively low and varies in a narrow range from 1.5 to 3.5 cases per 1,000 (Fig. 2). For instance, 10 Russian populations including Slav, Finno-Ugric, and North Caucasus populaces vary in their summed HD load from 1.59 per 1,000 in the cities of the Kirovsk region to 3.5 per 1,000 in rural Mari populations [53]. A similar variability of HD loads is observed in the native populations of Siberia [54, 55]. However, some forms of HD can vary in much wider intervals. For instance, the incidence of cystic fibrosis can vary up to 10-fold in different regions of Siberia [56].

In the case of HD, differences in disease incidence are directly linked to differences in the allele frequencies in the population. The main factors behind population dynamics, which form the overall picture of interpopulation differences in an HD load, are genetic drift and the founder effect [53]. Drift plays a leading role even when the size of the population is stable, and its effect is compounded by rapid changes in the effective population size (population waves). Overall, the role of natural selection in the HD gene differentiation of populations is small, since mutations that lead to HD lower the fitness of individuals, irrespective of ethnicity or geographical origins.

However, there are several interesting exceptions to this rule. The most well-known is the high number of individuals that are heterozygous for sickle-cell anemia and β-thalassemia in subtropical and tropical regions, such as the Mediterranean. Regions where these erythrocyte diseases occur frequently are virtually identical to those where malaria incidence is high [57]. Heterozygous carriers of the mutant alleles have a selective advantage due to their higher level of resistance to malaria, and the high frequency of heterozygotes is supported by balancing selection.

Another common hereditary disease, cystic fibrosis, occurs frequently among Europeans and is much less common in other geographical regions. The wide spread of the main mutation (ΔF508) across all of Europe and its rarity in other parts of the world indicate that this mutation appeared a long time ago, sometime after the migration of modern humans from Africa. Direct estimates of the mutation’s age using various methods suggest the opposite: namely that the mutation appeared relatively recently, about 10 thousand years ago [58, 59]. The likely reason for the wide spread of this mutation in Europe, just as for the erythrocyte diseases, is the lower susceptibility of ΔF508 heterozygotes to dehydration during typhoid and cholera, which remained a common menace in Europe until recently.

 Local adaptation of populations to dietary products can also lead to differentiation for certain diseases. Thus, the high incidence of the celiac disease in Northern Europe, especially in Scandinavian countries, and low incidence in Southern Europe are likely connected to the longer history of agriculture, specifically cereal plant cultivation, in the south of Europe and the prevalence of game as the main source of food for the Scandinavian population, which would mean that there were virtually no cereals in their diet. 

The role of genetic drift and the founder effect in the spread of HD can be illustrated well by the accumulation of certain forms of hereditary pathologies in some populations. Well-known examples of such populations are the Finns, Ashkenazi Jews, French Canadians and the Amish peoples. More than 20 so-called “Finnish” diseases have been documented - these are HD (mostly autosomal recessive), whose incidence among Finns is much higher than among other populations [60, 61]. The phenomenon of HD accumulation in the Finnish population is due to effective drift, long-term genetic isolation, and high occurrence of inbreeding. The same population mechanisms were probably the reason behind the accumulation of certain HD genes among the Ashkenazi Jews. They were also observed to have an extremely high incidence of more than 20 diseases (the most common among these being the Tay–Sachs disease and Type I Gaucher’s disease) [62].

In Russian populations, the phenomenon of ethnospecific diseases is most often seen in Yakuts (Table * 2* ). As many as 6 diseases can be termed “Yakut,” since the frequency of these diseases is above mean global frequencies by as much as several ten-fold. These diseases include two types of dwarfism which have been documented only recently [63, 64]. The population mechanism behind this accumulation of HD in Yakut groups is the founder effect, which coincided with some of the waves of expansion and migration of the Yakut people.

 Overall, some Mendelian HD can exhibit considerable interethnic frequency differences, which are due to differences in the frequency and type of mutations. These traits must, of course, be taken (and are taken) into account during medico-genetic counseling, DNA-diagnostics, and screening programs. In this context, DNA-diagnostics of HD can be considered as the first real use of personalized genomic medicine. 

**Genetic diversity and complex diseases. GWAS**

Interpopulation comparisons of the frequencies of common multi-factor diseases (MFD), also known as complex diseases, are complicated by the absence of homogenous medical statistics for global populations and the considerable clinical and genetic heterogeneity of MFD. However, there is a considerable amount of data on the interpopulation differences in the occurrence of MFDs, such as cardio-vascular diseases [68, 69], diabetes [70], some types of cancer [71], glaucoma [72], and nephropathies [73].

**Table 2:** Ethno-specific diseases in Yakuts

Diseases (OMIM number)	World prevalence (1 per 100,000)	Prevalence in Yakuts (1 per 100,000)	References
Spinocerebellar ataxia type 1 (164400)	1.0	38.6	[65]
Myotonic dystrophy (160900)	4.0-5.0	21.3	[66]
Inherited enzymopenic methaemoglobinaemia (250800)	1.0	5.7	[67]
Oculopharingeal muscular dystrophy (164300)	1.0	11.1	[63, 64]
3M syndrome (Yakut short stature syndrome) (273750)	25 cases	12.72	[64]
Syndrome of short stature with cone dysfunction, optic atrophy, and Pelger-Huet anomaly (SCOP) (not present in OMIM)	Not described	9.95	[64]

**Table 3:** Mortality rates in 4 major racial groups in U.S. ^1^

	Relative share in overall mortality inside the racial group				Mortality relative to White Americans^2^		
Cause of Death	Whites	African Americans	Latino Americans	Asians	African Americans	Latino Americans	Asians
Heart disease	27	26.6	25.3	25.5	0.98	0.94	0.94
Coronary heart disease	17.6	139	16.4	16.2	0.79	0.93	0.92
Stroke	52	6	5.7	8.6	1.15	1.09	1.65
Chronic obstructtive pulmonary diseases	4.9	26	2.5	2.8	0.53	0.51	0.57
Cancer	268	23.3	22.8	28.3	0.87	0.85	1.05
Pneumo-nia / Influenza	2.8	2.5	2.9	3.9	0.89	1.03	1.39
Liver diseases / Cirrhosis	1.6	1.1	3.5	0.9	0.69	2.18	0.56
Diabetes	2.7	4.2	5.5	3.3	1.55	2.03	1.22
HIV infection	0.6	3	1.8	0.3	5	3	0.5
External causes	10.4	9.9	13.4	9.2	0.95	1.29	0.88
All causes (per 100000)	450.4	690.9	332.8	264.6			

Notes. According to National Center for Health Statistics [74]. Mortality rate in White Americans is taken as 1.

The U.S. population can act as a good model for comparing the incidence of complex diseases: it is a multiracial country with a highly developed healthcare system and thorough medical statistics. Table 3 shows mortality data from the National Center of Statistics in Healthcare for the four main ethno-racial groups of the U.S. population - white Americans, African Americans, Hispanics from Latin America, and Asian Americans [74]. Since the overall mortality per 100,000 varies considerably (this value is twice higher for African Americans as compared to Latino Americans and Asians, while the mortality of the white population is intermediate), we recalculated these data as fractions of the overall value for each cause of death in each ethnic group. The right-hand side of the table lists ratios between the mortalities from various causes in three minor ethnic groups and white Americans. These data allow us to conclude that the two main causes of death in the U.S., namely cardiovascular and oncological diseases, which are responsible for half of the mortality rate, do not display any significant interracial differences. Other disease groups sometimes display considerable racial differences. Thus, the relative mortality due to diabetes is about 1.5 times higher for African Americans as compared to white Americans, while the mortality due to IHD (ischemic heart disease), chronic lung, and kidney diseases is much lower for African Americans. The relative mortality due to diabetes and kidney diseases among Hispanic Americans is more than twice as high compared with white Americans, while the mortality due to chronic lung diseases is twice as low. Asians die from strokes and pneumonia much more often than do white Americans; however, mortality due to COPD (Chronic Obstructive Pulmonary Disease) and kidney diseases is half as common in Asians as it is in white Americans.

 To what extent can such differences be attributed to interethnic genetic differentiation? Important information on this subject can be obtained by analyzing the associations of genetic markers with complex diseases, including genome-wide association searches. 

Ioannides *et al* . [75] compared the frequencies of genetic markers and their effects on diseases in the European, Asian, and African populations. They conducted a meta-analysis of 135 gene-disease associations, 45 of which proved to be statistically significant either on the level of a general meta-analysis (32 associations), or at least at the level of a single racial group (11 associations). The data of 45 meaningful meta-analyses encompassed 697 individual association studies with a combined cohort size of 300,000 individuals. The authors detected a statistically significant heterogeneity of the disease-associated allele frequencies (i.e. meaningful interpopulation differences) for 58% of the gene-disease associations. Significant differences in OR (odds ratio, a measure of the genetic risk of disease incidence) were detected only in 14% of the meta-analyses. Notably, interracial comparisons did not yield any significant associations with opposite effects in different populations.

 These data indicate that the differences in susceptibility gene frequencies may be one of the causes behind the interethnic differences in MFD incidence. However, the biological effect of the associated alleles is unidirectional, irrespective of the racial/ethnic origins, even though the relative share of the marker in the disease or susceptibility can vary. This is most probably due to the genotype (haplotype) surroundings, as well as gene-gene and gene-environment interactions. 

In recent years, the main source of new data concerning MFD susceptibility genes has been genome-wide association studies (GWAS). GWAS requires high-throughput analysis, which is achieved by using large cohorts (several hundreds or thousands) representative of the population, and a large number (hundreds of thousands) of tested polymorphisms, which are representative of the genomic diversity. A catalog of published GWAS is supported by the U.S. National Institute of Genomic Research and includes GWAS which were performed under very strict criteria: no fewer than 100,000 SNP must be analyzed, and the level of significance of a SNP-trait association must be no lower than 0.00001 [76]. As of the end of March 2010, the catalog contains 527 published studies and 2,516 SNP that are reliably associated with complex phenotypes.

A major part of these GWAS have been conducted on European populations, and no reliable estimations of the interethnic differentiation of disease-associated genome regions can be made using GWAS data alone. Adeyemo and Rotimi [77] managed to skirt this problem by analyzing the genetic heterogeneity of markers chosen from the GWAS catalog in populations from the HapMap project. The HapMap project (a map of human haplotypes) currently contains data on the polymorphism of several million SNP and the level of linkage disequilibrium across the whole genome for 11 populations of various ethnic origins, which are representative of the world population (Europeans, Asians, Africans, Indians, and Latin Americans). Adeyemo and Rotimi chose 621 SNPs from the GWAS catalog, which were associated with 26 complex diseases, including Alzheimer’s disease, hypertension, obesity, schizophrenia, type I and type II diabetes, rheumatoid arthritis, certain types of cancer, etc. The allelic frequencies of the chosen SNPs varied across the populations in a relatively wide range - differences of up to 20 to 40-fold were observed between some pairs of population groups. The interpopulation genetic diversity ratio ( *F*_st_ ) also varied considerably from marker to marker (such as, from 0.02 to 0.2 for type II diabetes or from 0.006 to 0.52 for the level of lipids). The mean level of interpopulation difference was 10.5%; i.e., it did not significantly differ from the differentiation level observed for conditionally neutral or genome-wide datasets of markers.

 These data suggest that the level of interpopulation and interethnic differences for genes associated with MFD does not differ from the general level of differentiation in the gene-pool, and that the risk of developing a disease associated with a genetic marker can vary significantly between population groups, depending on the frequencies of the associated marker and the modulating effects of other genes and environmental factors. 

The role of gene-gene and gene-environment interactions is usually hard to differentiate; however, their overall effect in the modification of disease risk associated with a certain gene or marker can be very significant and has population specificity. As an example, we can examine data for the role of the ε4 allele of the *APOE* gene in Alzheimer’s disease (AD). Reliable association of this marker with AD has been observed for all of the tested race groups; however, the frequency of the allele differs considerably (9% among the Japanese population, 14% among white Americans, 19% among African Americans). Homozygozity for the ε4 allele increases the risk of AD 33-fold for Japanese, 15-fold for white Americans, and only 6-fold for African Americans. For heterozygous individuals these values are correspondingly 5.6–3–1.1.

 Thus, even if the risk of complex disease development is reliably associated with distinct genetic markers in all populations and these markers have unidirectional effects, the magnitude of the effect or the severity of the risk still greatly depends on ethnicity- and population-specific factors of genetic and probably non-genetic nature. So, data on ethnic origins can provide additional information in making personalized medical prognoses. 

##  Ethnogenetics and pharmacogenomics 

One of the main advantages of personalized medicine is the individualization of drug therapy. Response to a drug, choice of the optimal drug class, dosage, and usage schedules can, at least partially, be determined using genetic factors. Information on individual genetic markers can help a clinician select the appropriate drug strategy. Based on these principles, pharmacogeneticists attempt to identify the genes and gene variants that influence the efficiency of drug therapy and lower the risk of side effects. It has been shown that the most widely used drugs are only effective in 25-60% of patients, and there have been 2 million cases of side effects per year in the U.S. alone, including more than a 100,000 deaths [78].

Pharmocogenomics studies have collected considerable data on the association of genetic markers with the effectiveness of certain drugs. During the last 20 years there have been approximately 2,000 studies on this subject, and hundreds of genes associated with drug therapy efficiency have been identified [79]. Most of these pharmacogenetics studies concern cardiovascular, oncological, and neurological diseases.

The FDA (Food and Drug Administration) of the U.S. Ministry of Healthcare has approved the addition of genetic marker information into the annotations of about 30 drugs, including warfarin, abacavir, imatinib, atorvastatin, etc. [80]. The list of biomarkers which were appended into these annotations includes genes that encode cytochromes, a low density lipoprotein receptor, N-acetyltransferases, epidermal growth factor receptor, etc.. The effects of drug therapy that depend on the genotypes for these markers include the clinical response to therapy, risk of side effects, choice of optimal dosage, sensitivity or resistance towards the drug, and polymorphism of drug targets.

Most of the relevant pharmacogenetic data has been collected on Caucasoids - more than 80% of the published research has been conducted on the European and U.S. populations [80], which is why there is little information on the interethnic differences of drug efficiency and on the role of genetic factors. As an example, we can note the decreased effectiveness of enalapril, weakened effect of the vasodilator sodium nitroprusside (an antihypertnesion vasodilator), and decreased effect of propranolol and atenolol (adrenoreceptor blockers) during hypertension therapy for African Americans as compared to Caucasians [81]. In some cases, the interethnic differences can be associated with frequency differences for a specific marker. Thus, the difference in propranolol and atenolol efficiency is due to the higher frequency of one of the missense mutations of the β1-adrenoergic receptor in white Americans (72%) as compared to African Americans (57%).

**Fig. 3:**
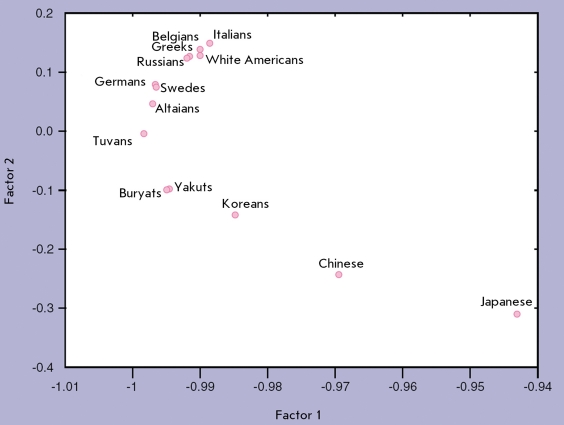
Geographic pattern of Russian and worldwide populations in the space of two first principal components of CYP gene frequencies (according to [83], with alterations).

We can surmise that interracial and interethnic differences in the effectiveness of drug therapy can be as common as interpopulation differences in MFD frequencies, since the genetic variability of genes that metabolize therapeutic drugs is the same as that of complex disease susceptibility genes [82, 83]. For instance, there is a 10-fold difference in the frequency of slow metabolizing variants of cytochrome CYP2D6 between Caucasians and Asians (10% for Caucasians and 1% for Japanese). This enzyme is involved in the metabolism of over 40 drugs, such as the widely used β-blockers and tricyclic antidepressants. The frequency of extremely fast metabolizing alleles of this enzyme exhibits a 10-fold difference even inside Europe - about 1-2% in Spain and up to 10% in Sweden. Our data also indicate a considerable variability within Russian populations in terms of drug metabolizing genes. For instance, the frequency of the CYP2C9*2 allele of one of the cytochrome genes is 12% for Russians, which is within the variability interval observed for Europeans (10–17%), while the allele is not present in Eastern Asian populations and occurs in the native populations of Siberia with a frequency of 1 to 6% [83]. The overall level of genetic differentiation for cytochrome genes is relatively low ( *F*_st _ = 0.021) in Russian populations; however, it is tightly correlated with the geographical layout, as are most other marker systems (Fig. 3).

##  From the human genome to the individual genome 

Re-sequencing of complete individual genomes yields new data on the genetic variability of humans and can help create individual health prognoses in the future. The first personal genome to be sequenced was the genome of Craig Venter, one of the key figures in the study of the human genome. Venter’s genome sequence was completed in October 2007 [84]. Data on the second sequenced genome (of Nobel Laureate James Watson) were published half a year later [85]. Currently (from the middle of 2010), there are data on 20 re-sequenced genomes (Table 4), which include those of Craig Venter, James Watson, archbishop Desmond Tutu, the twice-sequenced genome of a Yoruba individual, a Chinese genome, two Koreans, several Europeans, an ancient Eskimo, a Russian, and an Indian [84–99].

Apart from the complete genomes of unrelated healthy people of various descents and renown, researchers have also obtained complete sequences of patients with monogenic diseases: these include four genomes of a family quartet in which the children suffer from Miller syndrome (primary ciliary diskinesia in a lung form, which is phenotypically similar to cystic fibrosis) [98], a as well as a genome of a type I Charcot–Marie–Tooth disease patient [97]. No less than 7 complete tumor cell genomes have been published for cancers of various localizations: acute myeloid leucosis, malignant melanoma, glioblastoma, etc. [86, 100–105].

Progress in sequencing technology has been colossal in terms of speed and cost reduction. The sequencing of Venter’s genome, which was conducted on first generation sequencers, cost his company about 2 million U.S. dollars. Genomes which are sequenced on second-generation machines (Illumina Genome Analyzer and Applied Biosystems Solid System) cost about 200–500,000, while the most recent studies involving the re-sequencing of complete genomes cost no more than 1,500 U.S. dollars. So, Francis Colins’ [106] prognosis in 2001that the cost of sequencing a genome would fall to 1,000 dollars by 2030 has come to pass 20 years ahead of time!

The “1,000 genomes” project is aimed at obtaining complete and accurate genome sequences of 2,000 individuals from different populations of the main geographical regions of the world (Africa, Europe, Asia, and the Americas) [107]. During the first phase of the project, 180 samples from four HapMap populations (CEU, YRI, CHB, and JPT) were sequenced, albeit with only a few repetitions (2-8).

 What new knowledge do personal genomes add to the understanding of human genetic variability and what is their use in personalized medicine? First of all, researchers discover new genetic variants – each genome has about 2.5–4 million SNPs, several thousands of insertions/deletions and several hundreds or thousands of CNVs (Table 2). Most of these variants are being described for the first time. Re-sequencing data from complete genomes confirms the level of individual variability observed for the genomes sequenced in the “Human Genome” project - on average 3 million SNP from a 3 billion base genome yield differences in 1 nucleotide in a thousand. 

 The genomes of two individuals overlap for about half of the SNPs (Table 5). The degree of relation between the genomes is correlated with the genetic differentiation between the populations to which these genomes belong. The Yoruba genome is the farthest removed from others (38–45% overlapping SNPs), while the most related genomes are those of а Chinese and Korean (60–67% overlapping SNPs). 

**Table 4:** Personal genomes chronology

	Date of publication (submission)	Person	Journal	Institution (country)	Platform	Cove­rage	Number of SNPs (millions)	Notes
1.	2007, October (9 May 2007)	Craig Venter (m)	PloS Biology, [83]	J Craig Venter Institute (U.S.)	ABI 3730xl	7.5	3.47	$10 mln
2.	2008, April (3 December 2007)	James Due Watson (m)	Nature [84]	Baylor College of Medicine / 454 Life Sciences (U.S.)	Roche / 454	7.4	3.32	$2 mln
3.	2008, November (28 May 2008)	Caucasian American with acute myeloid leukemia (normal fibroblasts) (f)	Nature [85]	University of Washington (U.S.)	Illumina	13.9	2.92	$1 mln
4.	2008, November (24 June 2008)	Yoruba (NA18507) (m)	Nature [86]	Illumina / University of Cambridge (UK)	Illumina	41	3.45	$250,000
5.	2008, November (21 August 2008)	Chinese (Yanhuang, YH) (m)	Nature [87]	Beijing Genomic Institute (China)	Illumina	36	3.07	$500,000
6.	2009, May (3 February 2009)	Korean Seong-Jin Kim, SJK (m)	Genome Research [89]	Gachon University of Medicine and Science (Rep. of Korea)	Illumina	29	3.44	
7.	2009, June (1 February 2009)	Yoruba (NA18507) (m)	Genome Research [90]	Life Technologies (U.S.)	ABI SOLiD	17.9	3.87	
8.	2009, August (6 March 2009)	Korean, AK1 (m)	Nature [91]	Seoul National University (Rep. of Korea)	Illumina	27.8	3.45	
9.	2009, August (10 June 2009)	Steven Quake, Caucasoid USA, P0 (m)	Nature Biotechnol [92]	Stanford University (U.S.)	Helicos SMS Heliscope	28	2.81	Sequence from 1 molecule $48,000
10.	2009, December	Russian with kidney cancer (m)	Acta Naturae, [93]	RNC Kurchatov’s Institute (Russia)	Illumina/ABI SOLiD	16	?	
11.	2010, January (3 September 2009)	Caucasoid (NA07022) (m)	Science [94]	Complete Genomics (USA)	Complete Genomics DNA nanoarray	87	3.08	$8,000
12.	2010, January (3 September 2009)	Yoruba (NA19240) (f)	Science [94]	Complete Genomics (U.S.)	Complete Genomics DNA nanoarray	63	4.04	$3,400
13.	2010, January (3 September 2009)	Caucasoid (NA20431) (f)	Science [94]	Complete Genomics (U.S.)	Complete Genomics DNA nanoarray	45	2.91	$1,700
14.	2010, February (30 November 2009)	Paleo-Eskimo, Saqqaq (м)	Nature [95]	University of Copenhagen / Beijing Genomic Institute (Denmark / China)	Illumina	20	2.19	Ancient DNA (4000 years)
15.	2010, February (11 August 2009)	Khoisan, KB1 (m)	Nature [96]	Pennsylvania State University (U.S.)	Roche / 454 / Illumina	33.4	4.05	
16.	2010, February (11 August 2009)	Bantu, ABT Archbishop Desmond Tutu (m)	Nature [96]	Pennsylvania State University )	ABI SOLiD / Illumina	37.2	3.62	
17.	2010, March	Caucasoid, CMT1 patient (m)	New Eng J Med [97]	Baylor College of Medicine (U.S)	ABI SOLiD	29.9	3.42	
18.	2010, March (7 January 2010)	Pedigree #1, mother (Caucasoid, f)	Science [98]	Institute for System Biology / Complete Genomics (U.S.)	Complete Genomics DNA nanoarray	51	2.87	
19.	2010, March (7 January 2010)	Pedigree #1, father (Caucasoid, m)	Science [98]	Institute for System Biology / Complete Genomics (U.S.)	Complete Genomics DNA nanoarray	88	3.16	
20.	2010, March (7 January 2010)	Pedigree #1, daughter (Caucasoid, f, patient with Miller syndrome)	Science [98]	Institute for System Biology / Complete Genomics (U.S.)	Complete Genomics DNA nanoarray	54	3.23	
21.	2010, March (7 January 2010)	Pedigree #1, son (Caucasoid, m, patient with Miller syndrome)	Science [98]	Institute for System Biology / Complete Genomics (U.S.)	Complete Genomics DNA nanoarray	52	3.23	
22	2010, September (10 April 2010)	Irish	Genome Biol [99]	University College Dublin (Ireland)	Illumina	11	3.12	

**Table 5:** Characteristics of six individual genomes

Genome	Ethnic origin, Country	Number of SNPs	Overlapping with other genomes by common (%)					
			HuRef	Watson	NA18507	YH	SJK	Saqqaq
HuRef (Venter)	White American, U.S.	3075858	100	55.8	52.9	51.6	56.4	34.2
Watson	White American, U.S.	3321942	51.6	100	50.8	49.9	54.9	36
NA18507	Yoruba, Nigeria	4189457	38.8	40.3	100	42.1	45.8	27
YH	Chinese, China	3074097	51.6	54	57.3	100	67.3	38.2
SJK	Korean, South Korea	3439107	50.5	53	55.8	60.1	100	39
Saqqaq	Paleo-Eskimo, Greenland	2193396	47.9	33.9	32.5	53.5	61.1	100

**Fig. 4:**
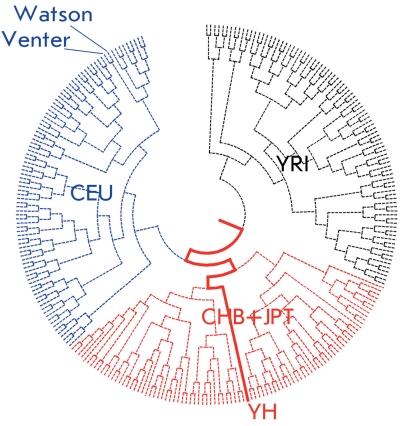
Individual genomes of Venter, Watson, and a Chinese individual (YH) on the tree of HapMap individuals (according to [88], with alterations).

 “Overlaying” the variable positions in complete genomes onto the data obtained in large-scale population projects (such as HapMap) allows us to glimpse at the genetic origins of an individual. For instance, by comparing SNPs in the Venter, Watson, and YH Chinese genomes with four HapMap populations (Fig. 4), based on the marker distributions in populations CEU, YRI, CHB, and JPT, researchers can estimate the level of cross-breeding between the main racial and ethnic components observed in these genomes. Thus, the shares of Caucasoid, Negroid, and Mongoloid components in Venter’s genome were estimated at 93.3, 5.1, and 1.6% respectively. The genome of the Nobel-prize winner Watson had a lower share of Caucasoid SNP (73.0%) due to the increased amount of Negroid markers (25.6%). 

 A personal genome sequence provides complete information on whether the individual carries any alleles associated with clinical phenotypes and is thus extremely valuable for individual health prognoses and for estimating MFD risks. The precision and relevance of the genetic health prognosis are thus unlimited by the technical possibilities of genome study but depend on the amount of knowledge on a phenome and its genetic determinants. 

 Data on complete genomes are insufficient at the moment in order to systematize the layout of interpopulation differences on a genome-wide scale; however, the existence of interindividual and interracial differences in the number of MFD-associated markers is obvious. Thus, Venter’s genome has about 50 SNPs which are associated with alcoholism. The sequenced Yoruba genome has 30, Watson - 25, and the Mongoloids (Chinese, Korean, and ancient Eskimo) have less than 20. Venter is also a record-holder in terms of SNPs associated with tobacco addiction (about 40 SNPs). The Chinese and ancient Greenlander have about 20 of these markers, while Watson’s genome, as well as the Korean and Yoruba genomes, has none. 

The most complete approach to using genome-scale information for making individual health prognoses was demonstrated in the recent work of Ashley *et al* . [108]. In order to estimate the risk of a certain disease, they proposed the use of a so-called pretest risk as a baseline. This pretest risk is an epidemiological risk estimate, which can in its simplest form be the incidence of this disease in the appropriate ethnic and age group. The individual genome was then tested for SNPs, which are reliably associated with the disease according to GWAS data. Based on these data, the researchers calculated post-test risk, which views each marker as an independent risk factor. An example calculation of the individual risk of myocardial infarction is shown in Table 6. The risk was estimated for a healthy 40-year-old white American, whose genome was sequenced in study [92]. The DNA donor had a family history of cardio-vascular diseases and some cases of sudden death among relatives. His biochemical and electrocardiogram parameters were normal. The pretest risk was estimated at 2%. The individual’s genome contained 7 SNPs which were reliably associated with myocardial infarction according to GWAS data. The OR (genetic risk estimate) values of the individual’s genotypes varied from 0.75 to 2.86. The overall risk of myocardial infarction (product of the pretest risk and OR of each marker) was 8.9%. In this case, the genetic composition of the tested individual increased the overall risk by 4.5-fold as compared with the pretest risk.

**Table 6:** Individual calculation of myocardial infarction risk based on genomic data (according to [108])

Gene	SNP	Genotype	OR	Risk, %
				Pretest
				Post-test
*LPA*	rs3798220	CT	1.86	3.7
*THBS2*	rs8089	AC	1.09	4.0
*LDLR*	rs14158	GG	2.88	10.6
*LIPC*	rs11630220	AG	1.15	12.0
*ESR2*	rs1271572	CC	0.73	9.1
*ESR2*	rs35410698	GG	1.03	9.4
*FXN*	rs3793456	AA	0.94	8.9

##  Conclusion 

 Humans display a relatively low level of genetic variability (both at the level of population differentiation and at the level of individual genomes), which is set on a background of high phenotypic variability and strict geographical structure of the genetic variation, which is manifested in the clustering of geographically adjacent populations. The spatial nature of the genetic variability distribution of modern humans can be observed at different levels of population structure and in various groups of markers, including the genes associated with MFD development. Genetic differences between human populations are responsible for only 10-15% of the genetic variability in humans. However, these differences prove significant in the field of personalized medicine in terms of diagnosing monogenic diseases, estimating the susceptibility to common diseases, and making health prognoses, and the efficiency of drug therapy. 

 Returning to the issues stated in the introduction, let’s sum up our current knowledge. Genetic differentiation in populations for disease genes is as large as the overall level of interpopulation diversity on a genome-wide scale. Observed interethnic differences in the incidence of human diseases can be almost completely (for Mendelian diseases) or to a significant degree (for MFD) explained by the different frequencies of disease-associated genes. The general “geographical pattern” of genetic diversity took shape during the spread of modern humans through migrations, genetic drift, and sudden changes in the effective size of populations. However, natural selection could have played a significant role in the development of the variability of some regions of the genome both on a global scale (such as the immune response or skin pigmentation gene) or at the level of population adaptations to the local environment (such as genes for metabolizing compounds present in the diet). The biological effects of distinct genetic variants (mutations of polymorphisms) in relation to disease are usually stable and do not depend on the racial, ethnic, or geographic context. However, the magnitude of the effects (the relative role of the marker in the disease itself or in the risk of disease development) can exhibit strong variation at the population and individual levels due to the different genetic (haplotypic) environment and modifying gene-gene and gene-environment interactions. 

Undoubtedly, the “ethnic factor” must be taken into account during medical studies, especially those concerning personalized genomic medicine. The long history of discussions on this issue in medical and genetic literature continues (see [36, 109–111]). However, the professional community of researchers who work in the field of personal genomics devotes considerable attention to population aspects both at the stage of data collection (such as the GWAS or pharmacogenomic studies) and at the data-interpretation stage of individual genetic testing or genetic screening at the population level [110].

 Integration of genomics and phenomics in the framework of system biology, novel powerful instruments for describing and analyzing genetic diversity - sequencing individual genomes and genome-wide analysis of SNP on microarrays, the HapMap and “1000 genomes” projects - all of these new happenings give hope as to fast progress in the categorization of genetic diversity associated with the risk of common diseases, the efficiency of drug therapy, and the establishment of a tight link between basic scientific results and proven recommendations for personalized medicine. 

## Abbreviations

ADD: autosomal dominant diseases
ARD: autosomal recessive diseases
HVSI: hypervariable segment I
mtDNA: mitochondrial DNA
CD: common diseases
RFLP: restriction fragments length polymorphism
IHD: ischemic heart disease
COLD: chronic obstructive lung disease
SNP: single nucleotide polymorphism
CNV: copy number variation
STR: short tandem repeats
HapMap: Haplotype Map of the Human Genome
CEU: population of Central European origin
YRI: population of Yoruba from Ibadan, Nigeria
CHB: Chinese population from Beijing
JPT: Japanese population from Tokyo
CMT1: Charcot-Marie-Tooth disease, type 1
GWAS: genome-wide associations search
OR: odds ratio
OMIM: on-line Mendelian Inheritance in Man catalogue
